# What Are People Tweeting About Zika? An Exploratory Study Concerning Its Symptoms, Treatment, Transmission, and Prevention

**DOI:** 10.2196/publichealth.7157

**Published:** 2017-06-19

**Authors:** Michele Miller, Tanvi Banerjee, Roopteja Muppalla, William Romine, Amit Sheth

**Affiliations:** ^1^ Department of Biological Sciences Wright State University Dayton, OH United States; ^2^ Department of Computer Science and Engineering Wright State University Dayton, OH United States; ^3^ Kno.e.sis Computer Science and Engineering Wright State University Dayton, OH United States

**Keywords:** viruses, epidemiology, social media, machine learning

## Abstract

**Background:**

In order to harness what people are tweeting about Zika, there needs to be a computational framework that leverages machine learning techniques to recognize relevant Zika tweets and, further, categorize these into disease-specific categories to address specific societal concerns related to the prevention, transmission, symptoms, and treatment of Zika virus.

**Objective:**

The purpose of this study was to determine the relevancy of the tweets and what people were tweeting about the 4 disease characteristics of Zika: symptoms, transmission, prevention, and treatment.

**Methods:**

A combination of natural language processing and machine learning techniques was used to determine what people were tweeting about Zika. Specifically, a two-stage classifier system was built to find relevant tweets about Zika, and then the tweets were categorized into 4 disease categories. Tweets in each disease category were then examined using latent Dirichlet allocation (LDA) to determine the 5 main tweet topics for each disease characteristic.

**Results:**

Over 4 months, 1,234,605 tweets were collected. The number of tweets by males and females was similar (28.47% [351,453/1,234,605] and 23.02% [284,207/1,234,605], respectively). The classifier performed well on the training and test data for relevancy (F1 score=0.87 and 0.99, respectively) and disease characteristics (F1 score=0.79 and 0.90, respectively). Five topics for each category were found and discussed, with a focus on the symptoms category.

**Conclusions:**

We demonstrate how categories of discussion on Twitter about an epidemic can be discovered so that public health officials can understand specific societal concerns within the disease-specific categories. Our two-stage classifier was able to identify relevant tweets to enable more specific analysis, including the specific aspects of Zika that were being discussed as well as misinformation being expressed. Future studies can capture sentiments and opinions on epidemic outbreaks like Zika virus in real time, which will likely inform efforts to educate the public at large.

## Introduction

### Background

The 2014 and 2015 Ebola outbreak caused fear and misinformation to spread wildly across the globe. It was shown that the spread of misinformation led to deaths due to improper practice of appropriate preventative measures [1].

Experts at the Center for Disease Control (CDC) and the World Health Organization (WHO) admit that they mishandled the response for Ebola by not responding to the threat sooner [2]. One year after the Ebola outbreak ended, the Zika outbreak started and also caused fear and misinformation to spread. In the recent years, citizen sensing has picked up greatly with the rise of mobile device popularity and social media sites such as Facebook and Twitter. The idea with citizen sensing is that citizens play the role of sensors in the environment [3], providing information regarding health care issues such as disease outbreaks like Ebola and Zika [4].

Big social data eliminate the time lag caused by traditional survey-based methods, allowing for studying public opinions on issues while addressing privacy concerns of users through group-level analyses of public behavior with respect to specific issues in real time. In particular, public opinion mining has facilitated exploration of public views on important social issues such as gender-based violence [5] and health-related beliefs [6-7].

With respect to Zika, Twitter served as a source of misinformation. To counter, the CDC responded with correct information, either by tweeting general statements about Zika or by responding to questions and comments directed at them. For example, 1 user tweeted, “Apparently Florida is immune to the Zika virus,” whereas the CDC had tweeted about Zika in Florida several times, including this tweet: “Updated: CDC travel and testing recommendations for Miami-Dade county b/c of continued local #Zika transmission.”

### Zika

Many people do not even realize they are sick from Zika, let alone the need to go to the hospital; and death due to Zika is extremely rare [8]. The Zika virus usually causes only mild symptoms such as headache, rash, fever, conjunctivitis, and joint pain, which can last from a few days to a week after being infected [8]. Guillain-Barré syndrome and microcephaly have been linked to Zika and as this is the first outbreak of Zika associated with these defects, management is still an important challenge [9]. There are 3 main ways by which one can contract Zika: (1) being bitten by an infected Aedes mosquito, (2) through sexual contact, and (3) from mother to fetus [8]. There is currently no medicine or vaccine to treat the Zika virus; however, there are several methods of prevention [8].

### Related Works

A study by Oyeyemi et al [10] concerning misinformation about Ebola on Twitter found that 44.0% (248/564) of the tweets about Ebola were retweeted at least once, with 38.3% (95/248) of those tweets being scientifically accurate, whereas 58.9% (146/248) were inaccurate. Furthermore, most of the tweets containing misinformation were never corrected. Another study about Ebola by Tran and Lee [4] found that the first reported incident of the doctor with Ebola had more impact and received more attention than any other incident, showing that people pay more attention and react more strongly to a new issue.

Majumder et al attempted to estimate the basic R_0_ and R_obs_ for Zika using HealthMap and Google Trends [11]. R_0_ is known as the basic reproduction number and is the number of expected new infections per first infected individual in a disease-free population. R_obs_ is the observed number of secondary cases per infected individual. Their results indicate that the ranges for R_obs_ were comparable between the traditional method and the novel method. However, traditional methods had higher R_0_ estimates than the HealthMap and Google Trend data. This indicates that digital surveillance methods can estimate transmission parameters in real time in the absence of traditional methods.

Another study collected tweets on Zika for 3 months [12]. They found that citizens were more concerned with the long-term issues than the short-term issues such as fever and rash. Using hierarchical clustering and word co-occurrence analysis, they found underlying themes related to immediate effects such as the spread of Zika. Long-term effects had themes such as pregnancy. One issue with this paper was that they never employed experts to check the relevance of the tweets with respect to these topics, which is a common problem in mining social media data.

A study by Glowacki et al [13] collected tweets during an hour-long live CDC twitter chat. They only included words used in more than 4 messages to do a topic analysis and found that the 10-topic solution best explained the themes. Some of the themes were virology of Zika, spread, consequences for infants and pregnant women, sexual transmission, and symptoms. This was a curated study where only tweets to and from the CDC were explored, whereas the aim of our larger study was to determine what the general public was discussing about Zika.

A study by Fu et al [14] analyzed tweets from May 1, 2015 to April 2, 2016 and found 5 themes using topic modeling: (1) government, private and public sector, and general public response to the outbreak; (2) transmission routes; (3) societal impacts of the outbreak; (4) case reports; and (5) pregnancy and microcephaly. This study did not check for noise within the social media data. Moreover, the computational analysis was limited to 3 days of data, which may not reflect the themes in the larger dataset.

In many of these studies, the need for checking the performance of the system as well as a post hoc error analysis on checking for the generalizability of their method is overlooked. We address this in our study by employing machine learning techniques on an annotated data set, as well as a post hoc error analysis on a test dataset, to ensure the generalizability of our system.

**Figure 1:**
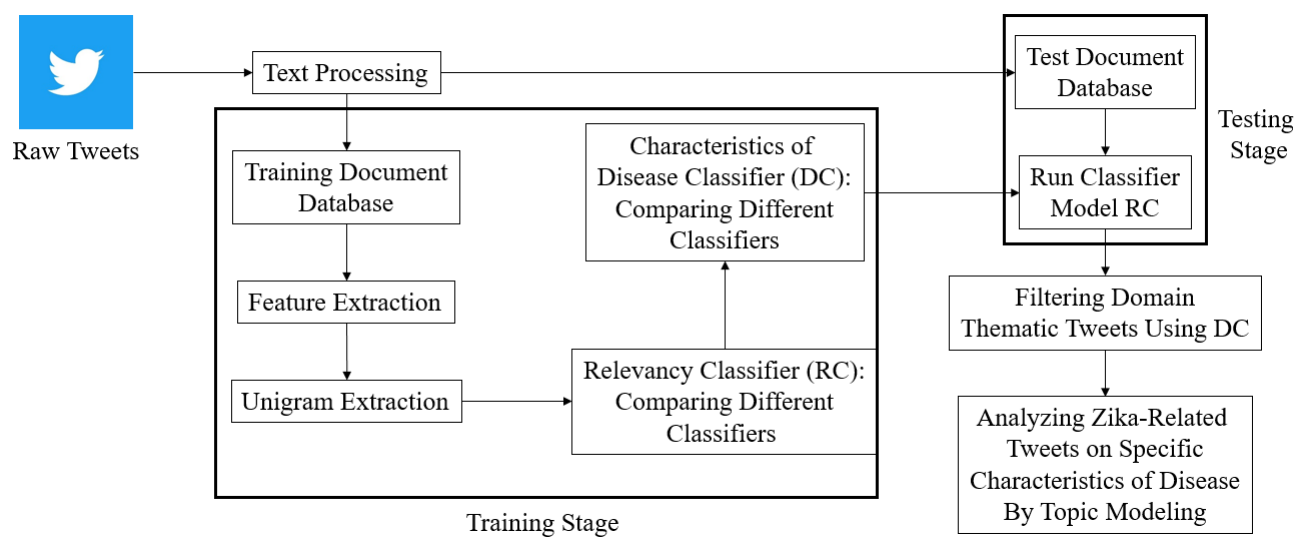
Block diagram of the pragmatic function-oriented content retrieval using a hierarchical supervised classification technique, followed by deeper analysis for characteristics of disease content.

In this study, an exploratory analysis focused on finding important subcategories of discussion topics from Zika-related tweets was performed. Specifically, we addressed 4 key characteristics of Zika: symptoms, transmission, treatment, and prevention. Using the system described in Figure 1, the following research questions were addressed:

*R1.* Dataset Distribution Analysis: What proportion of male and female users tweeted about Zika, what were the polarities of the tweets by male and female users, and what were the proportions of tweets that discussed topics related to the different disease characteristics—symptoms, transmission, treatment, and prevention?

*R2.* Classification Performance Analysis: What was the agreement among annotators’ labels that were used as the ground truth in this study, what was the classification performance to detect the tweets relevant to Zika, and how well were the classifiers able to distinguish between tweets on the different disease characteristics?

*R3.* Topical Analysis: What were the main discussion topics in each of these categories, and what were the most persistent concerns or misconceptions regarding the Zika virus?

## Methods

In this exploratory study, a combination of natural language processing and machine learning techniques was used to determine what information about Zika symptoms, transmission, prevention, and treatment people were discussing on Twitter. Specifically, a 2-stage classifier system was built for finding relevant tweets on Zika and then categorizing these into 4 disease categories: symptoms, transmission, prevention, and treatment (Figure 1).

### Dataset Distribution (Addressing R1)

#### Data Collection

Tweets were collected between February 24, 2016 and April 27, 2016 for a total of 1,234,605 tweets using Twitris 2.0 [15]. During this time frame, a lot of people were tweeting about their concern about hosting the Olympics, new information about Zika was being found weekly, and it was right after Zika was linked to microcephaly and Guillain-Barré syndrome. We used a streaming application program interface (API) from the Twitris system [15] to collect the tweets, which means we only had access to a small percent of the tweets. We initially started the search using only the keyword “Zika” but quickly realized that the search was capturing a large number of tweets unrelated to Zika virus. We then created a semantic concept called Zika that utilized 2 terms “Zika” and “Zika virus,” which improved the quality of tweets for the data collection. This may be due to the fact that the Twitter streaming API allows collection of around 1% of the total tweets streaming at a given time [16]. Finally, the keyword “treatment” was added to the Zika concept as there were hardly any tweets about treatment, which was not surprising because there is currently no treatment for Zika. Adding the keyword “treatment” allowed us to check for social media responses to the significant drug and vaccine research being implemented during the time of tweet collection. The other disease-related category titles (prevention, transmission, and symptoms) did not need to be included as keywords as we observed that more than enough tweets were being collected for those categories.

#### Labeling Process and Data Annotation

Three microbiology and immunology experts annotated 1467 random tweets as being relevant or nonrelevant. Tweets were considered relevant if it contained information about Zika and the focus of the tweet was on Zika. For example, “Millions of GM mosquitoes to fight Zika virus in Caymans” was annotated as relevant as the tweet is about using genetically modified (GM) mosquitoes to fight Zika, whereas “#MoreTrustedThanHillary going to Brazil during Zika virus season” was annotated as nonrelevant as the focus of the tweet is on making fun of Hillary Clinton and is not about Zika. The relevant tweets were then further categorized as pertaining to the topic of (1) symptoms, (2) treatment, (3) transmission, or (4) prevention by the same 3 experts. Tweets were categorized as “symptoms” if they pertained to any of the symptoms associated with Zika as seen in this tweet: “WHO sees scientific consensus on Zika virus as cause for disorders.” Tweets were categorized as “treatment” if they mentioned the fact that there is no treatment, research related to treatments, or included information about fake treatments. Here is an example of a treatment tweet: “Zika virus cloned in step toward vaccine.” Tweets were categorized as “transmission” if they mentioned modes of transmission, mosquitoes, or the Olympics. Here is an example of a transmission tweet: “Zika virus strain responsible for the outbreaks in Brazil has been detected in Africa.” Finally, tweets were categorized as “prevention” if they discussed ways to prevent the spread of Zika, or funding to fight Zika. Here is an example of a prevention tweet: “Senate Nears Deal for at Least $1.1 Billion to Fight Zika Virus.” These 4 categories were used because they are characteristics of disease used in many medical journals and by the CDC and WHO. Fleiss kappa [17] was used to quantify the interrater reliability of our expert annotators.

#### Preprocessing

Before analysis, the data were preprocessed to remove the URL, screen handles (@username), retweet indicators, and non-ascii characters. Data were further normalized by removing capital letters, numbers, punctuations, and whitespaces from the tweets. Terms were filtered out to remove single characters like “d,” “e,” which do not convey any meaning about the topics in the corpus, and top words like “and,” “so,” etc were removed for the classification stage. Each tweet was represented as a feature vector of the words present in the tweet using unigrams.

### Classification Performance (Addressing R2)

Supervised classification techniques including the decision tree (J48), multinomial Naive Bayes (MNB), Bayesian networks (Bayes Net), sequential minimal optimization (SMO) using support vector machine (SVM), Adaboost, as well as bagging or bootstrapping (Bagging) techniques were implemented on the Zika dataset for (1) classifying whether a tweet was relevant or nonrelevant, and (2) if relevant, further categorizing the tweets into the disease characteristics. Supervised techniques rely on labeled data, in this case tweets that are manually labeled as relevant to Zika virus, as well as the category it belongs to: Zika symptoms, Zika treatment, Zika transmission, and Zika prevention. They “learn” the nature of the tweets in the different groups and subgroups.

The performance of each classifier was assessed using the tenfold cross-validation, which is a commonly used method for the evaluation of classification algorithms that diminishes the bias in the estimation of classifier performance [18]. This approach uses the entire dataset for both training and testing, and is especially useful when the manually labeled dataset is relatively small. The study reports the average of the precision, recall, F-scores, and area under the curve (AUC) as measures of classification performance.

### Topical Analysis (Addressing R3)

Studies such as Hong and Davison [19] have shown the utility of using traditional topic modeling methods like latent Dirichlet allocation (LDA) for grouping of themes occurring in short text documents. The basic idea in LDA is that documents (tweets in this case) are represented as random mixtures over hidden topics, where each topic is characterized by a distribution over words that occur most frequently within that topic [20]. In this study, we use topic modeling for finding the underlying topics in each of the 4 disease characteristics to facilitate more detailed qualitative exploration of the types of discussions that occur within each disease characteristic.

Perplexity is a common measure to evaluate the topic models generated by LDA [21]. We use this measure to evaluate the topic modeling results by testing out different numbers of topic models from 2 to 20 for all 4 disease categories—symptoms, transmission, prevention, and treatment—using the well-established 10-fold cross-validation technique to ensure repeatability as well as generalizability.

## Results

### Dataset Distribution (Addressing R1)

**Figure 2:**
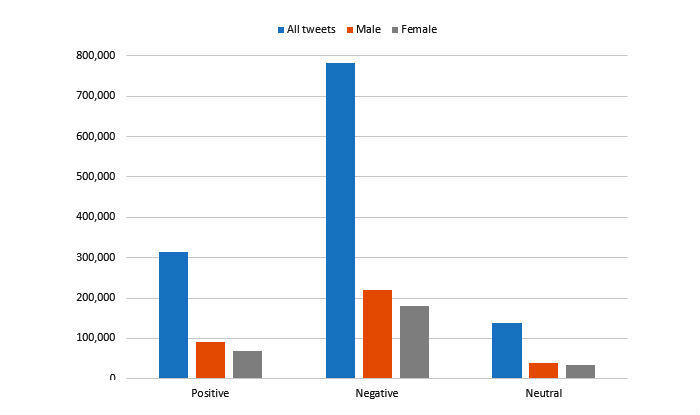
Polarity and proportion of tweets divided in the gender categories.

**Figure 3:**
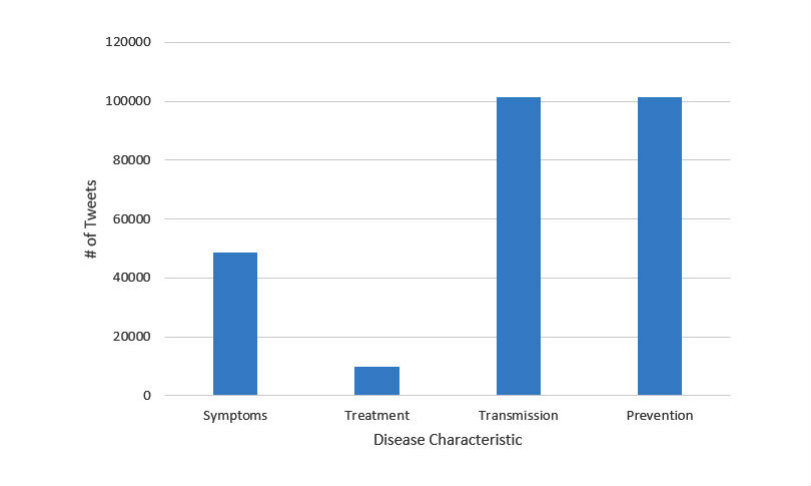
Number of tweets in each disease category after classifying all tweets (1.2 million tweets) using the best classification model multinomial Naive Bayes (discussed in the Classification and Performance Using 10-fold Cross-Validation section).

Overall, 41.88% (517,070/1,234,605) of tweets contained a retweet and 84.60% (1,044,489/1,234,605) contained a URL. Tweets by gender were found by twitter usernames using the genderize API [22]. According to genderize, 28.47% (351,453/1,234,605) of the tweets were by males, 23.02% (284,207/1,234,605) by females, and 48.51% (598,945/ 1,234,605) were by unknown gender. The polarity of the individual tweets was found using the sentiment package in R [23] (Figure 2). The polarity of the tweets between males and females was similar. We further found a class imbalance in the categories (Figure 3). As there is no treatment for Zika, not many people tweeted about it. Transmission and prevention tweets were most frequent, indicating that they were the most discussed topics concerning Zika.

### Classification Performance (Addressing R2)

**Figure 4:**
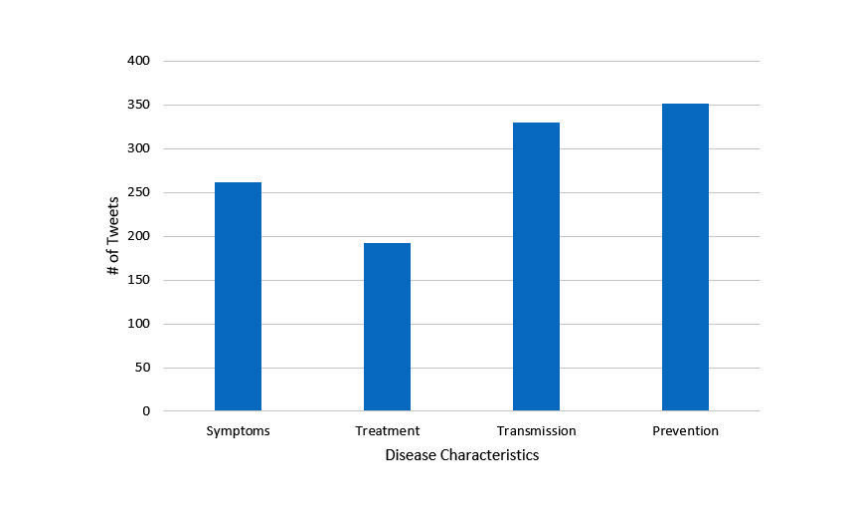
Number of tweets from the labeled dataset for each of the 4 categories of disease characteristics.

In the first stage of the categorization process for the ground truth tweets, tweets were first classified as being relevant or not relevant to Zika. Tweets that were relevant were then categorized as being about symptoms, treatment, transmission, or prevention. To train the classifiers and evaluate their performance, 1467 tweets were manually labeled. Figure 4 provides the distribution of the relevant tweets in the 4 categories. As seen from Figure 3, the distribution of the labeled gold standard dataset was similar to the distribution of the large data corpus, except for a larger portion of tweets related to treatment.

**Table 1:** Different classifier performances for detecting relevant tweets using decision tree (J48), multinomial Naive Bayes (MNB), Bayesian networks (Bayes Net), sequential minimal optimization (SMO) using support vector machine (SVM), and bagging or bootstrapping (Bagging) techniques.

Classifier	TP^a^	FP^b^	Precision	Recall	F1 score	AUC^c^
J48	0.821	0.390	0.812	0.821	0.815	0.784
MNB (bayes)	0.880	0.368	0.881	0.880	0.868	0.943
Bayes Net	0.832	0.479	0.821	0.832	0.812	0.837
SMO	0.895	0.252	0.892	0.895	0.892	0.822
Bagging	0.857	0.411	0.852	0.857	0.843	0.877

^a^TP: true positive.

^b^FP: false positive.

^c^AUC: area under the curve.

#### Interrater Reliability

Fleiss kappa values for relevant or not was .71. Fleiss kappa values for symptoms, treatment, transmission, and prevention were .93, .62, .92, and .87, respectively. This indicates substantial to almost perfect agreement among the raters [24]. Given substantial interrater reliability, a model needed to be built based on the gold standard dataset.

#### Classification and Performance Using Tenfold Cross-Validation

Table 1 gives the performance of different classifiers on the 1467 preprocessed Twitter data to find the relevancy of the tweet toward Zika. Unigram features were extracted from the texts using the Weka toolbox [25]. For this dataset, the classifiers performed well, with AUC values ranging from 0.78 to 0.94. MNB outperformed other classifiers based on the F-measure (0.86) and AUC (0.94) (Table 1). MNB classifiers perform better for data sets that have a large variance in document length (in this case, the length of the tweets) by incorporating the evidence of each appearing word into its model [26].

The class imbalance was affecting the classifier performance. Although the AUC value was high (0.94), the classifier predicted a tweet was relevant more often than not relevant as 77.44% (1136/1467) of the tweets belonged to the relevant category.

Table 2 gives the performance of different classifiers on 1135 preprocessed Twitter data to find the categorical classification (symptoms, treatment, transmission, and prevention) of the tweets. Again, the classifiers performed well with AUC values ranging from 0.83 to 0.94. With this dataset, MNB outperforms other classifiers again.

**Table 2:** Different classifier performances for detecting the 4 disease categories within the relevant tweets using decision tree (J48), multinomial Naive Bayes (MNB), Bayesian networks (Bayes Net), sequential minimal optimization (SMO) using support vector machine (SVM), as well as bagging or bootstrapping (Bagging) techniques.

Classifier	TP^a^	FP^b^	Precision	Recall	F1 score	AUC^c^
J48	0.694	0.122	0.702	0.694	0.695	0.838
MNB	0.784	0.084	0.787	0.784	0.785	0.940
Bayes Net	0.697	0.121	0.729	0.697	0.702	0.885
SMO (SVM)	0.775	0.088	0.780	0.775	0.777	0.877
Bagging	0.727	0.112	0.741	0.727	0.730	0.901

^a^TP: true positive.

^b^FP: false positive.

^c^AUC: area under the curve.

**Table 3:** Precision, recall, and F-measure for each of the 4 disease characteristics.

Category	Symptoms	Treatment	Transmission	Prevention	Average
precision	0.98	0.97	0.86	0.94	0.94
Recall	0.81	0.97	0.88	0.83	0.87
F1 score	0.89	0.97	0.87	0.88	0.90

On the basis of the above results, the 2-stage classifier system was found to have a high precision and recall performance for categorizing the tweets into relevant and not relevant, and further classifying the relevant tweets into the 4 disease categories. Once the performance of the model based on the gold standard dataset was confirmed to have high precision and recall, the model needed to be tested on a new set of tweets.

#### Classification and Performance Based on Error Analysis Using Hold-Out Dataset

As a post hoc analysis of generalizability, 530 new tweets (also known as hold-out data) that were not included in the gold standard data set were analyzed using the 2-stage classifier model. High precision and recall values were obtained for the relevance classifier, with Precision =0.99 and Recall =0.99. Hence, the F-measure was also 0.99 (harmonic mean of precision and recall). This high performance of the classifier indicates that the gold standard dataset was a good representation of the distribution of the tweets in the large data corpus.

Even though the classes were unbalanced, high precision and recall values were still obtained for disease characteristics (Table 3) in the second classification stage. An overall high F-measure of 0.9 was obtained. This further indicated that the gold standard dataset was a good representation of the tweets, as well as the disease categories in the larger corpus.

The error analysis indicates that the classifiers performed well with the unseen test data and were generalizable enough to work with the large dataset. The dataset was further examined with a focus on the insights provided within each disease category. More specifically, the topics discussed on Twitter in the symptoms category were examined to discover the latent semantic topics discussed therein. The symptoms category was chosen because the researchers felt it was the topic of most concern due to all the defects associated with Zika.

### Topical Analysis (Addressing R3)

**Figure 5:**
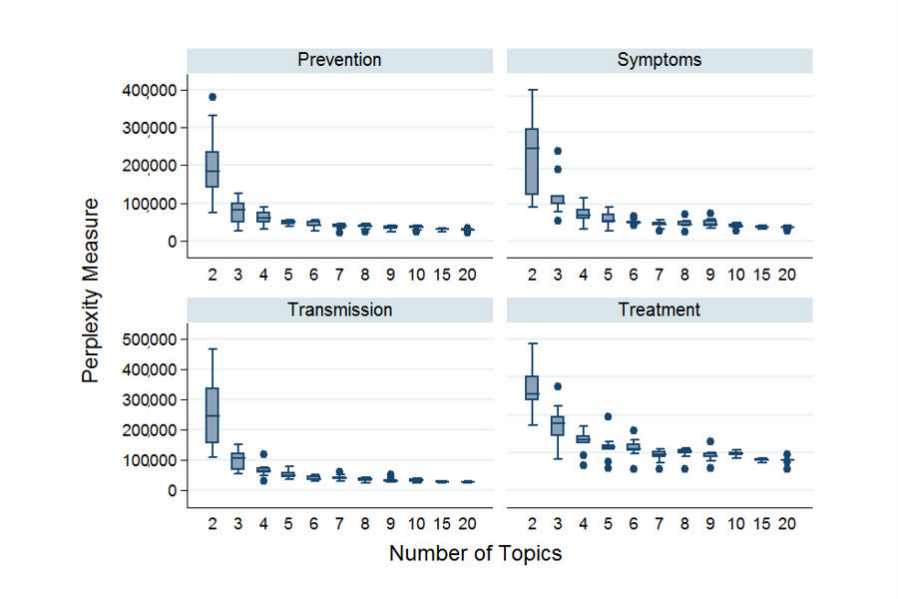
Prevention, symptoms, transmission, and treatment perplexity measure plots.

From Figure 5, we observe that the perplexity values decrease rapidly until about 5, and then level off after 5 for all the 4 categories, indicating that increasing the number of topics after 5 does not significantly improve the performance of the LDA models (the lower the perplexity value the better). Therefore, the number of topics was restricted to 5 while discussing the topics for each category.

The results of LDA are discussed for each of the 4 disease characteristics in Tables 4-5. Topic modeling results are shared here [27] for the research community to examine the outcome of using topic modeling, as well as the overlap among the topics generated. First, the results for the 3 categories, that is, prevention, transmission, and treatment, will be discussed. Then, a more detailed analysis of the topic modeling results for the “symptoms” category will be discussed along with the misinformation tweeted by users within that domain.

Table 4 provides the topics for the 3 categories: (1) prevention, (2) transmission, and (3) treatment, along with representative tweets within each topic.

Prevention: Within the prevention topics, topic #1 was *need to control and prevent spread*, topic #2 was *the need for money to combat mosquitoes and research treatments*, topic #3 was *ways to actually prevent spread*, topic #4 was *introducing a bill to get funds*, and topic #5 was *research* (Table 4).

Transmission: In transmission, there was a strong overlap in topics #1 (vector, ie, mosquitoes for Zika) and #4 (disease spread) that highlight the overlap between spread by mosquitoes and the spread of disease in general. Another topic (#2) was *sexual spread*, which is another mode of transmission besides through mosquitoes. The next topic (#3) was *infants*, who are most affected by this epidemic due to the risk of microcephaly. The final topic (#5) was *sports*.

Treatment: There was a slight overlap between topics #1 (lack of treatment) and #3 (vaccine development) primarily due to the large co-occurrence of the word “vaccine” in both these topics. *Blood testing* (#4) was another major topic as some people got infected with Zika after receiving a blood transfusion. As no treatment exists, a lot of research is focused on developing a drug for Zika, which is why *test development* (#5) was the final topic.

**Figure 6:**
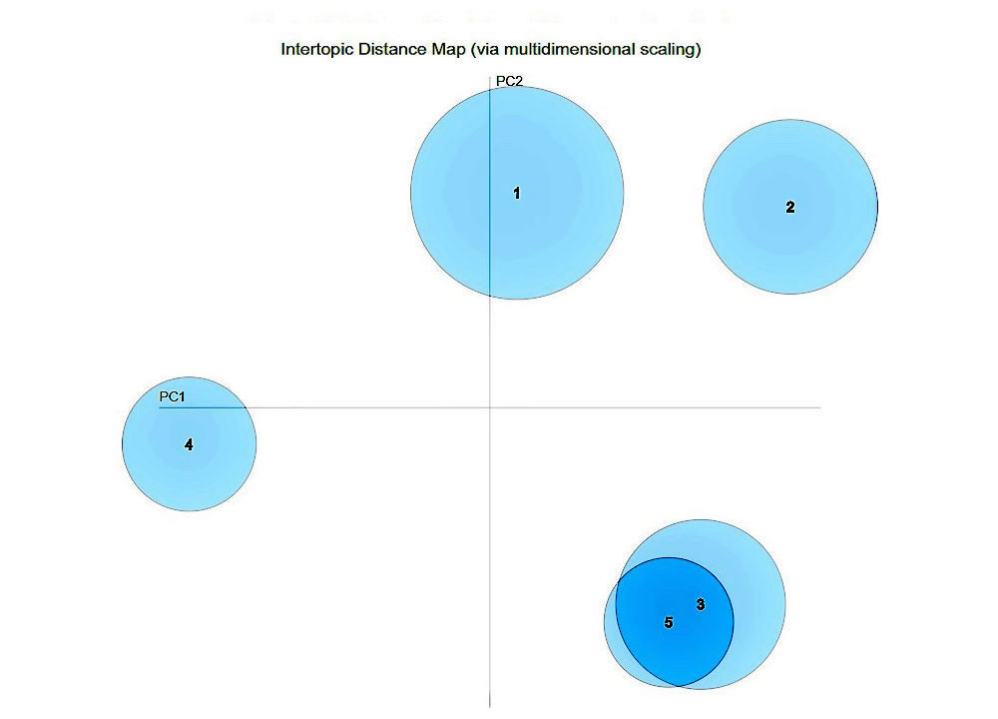
A 2-dimensional principal components plot of topics discussed pertaining to Zika symptoms.

Symptoms: In the topic model results for symptoms, topics #1 (zika effects), #2 (brain defects), and #4 (zika scarier than thought) were well separated, whereas topics #3 (confirmation of defects) and #5 (initial reports) overlap significantly (Figure 6 and Table 5). The topics are described in Table 5. Topics #3 and #5 overlap for symptoms because a lot of the initial reports for different locations were about new cases of microcephaly in that location as seen in this example tweet: “Colombia Reports First Cases of Microcephaly Linked to Zika Virus.” Topic #3 more strongly addressed the defects that were confirmed, whereas topic #5 focused on where reports came from.

In this section, the topic modeling results generate insightful results that allow researchers to understand the citizens’ concerns, as well as the spread of misinformation. According to the theory of LDA, each topic represents certain common properties that reflect the pattern in the tweets. Finding out the exact meanings of the topics requires additional information and domain knowledge. We see that for each of the disease characteristics, the discovered topics can be interpreted straightforwardly through the lens of domain-specific knowledge about Zika.

**Table 4:** Prevention, transmission, and treatment topic modeling results.

Disease characteristic	Topic	Sample tweets for each topic
Prevention	(#1) Control	RT^a^ @DrFriedenCDC: A2. The best way to prevent #Zika & other diseases spread by mosquitoes is to protect yourself from mosquito bites. #Reut
	(#2) Money need	#healthy Congress has not yet acted on Obama’s $2 billion in emergency funding for Zika, submitted in February
	(#3) Prevention	RT @bmj_latest: Couples at risk from exposure to Zika virus should consider delaying pregnancy, says @CDCgov
	(#4) Bill	https://t.co/Ke12LOdypf Senate Approves $1.1 Billion In Funding To Fight The Zika Virus #NYCnowApp
	(#5) Research	Florida is among those at greatest risk for Zika. @FLGovScott’s sweeping abortion bill blocks scientists’ access to conduct research
Transmission	(#1) Vectors (mosquitoes)	This map shows the Northeast is at risk for Zika mosquitoes this summer
	(#2) Sexual	@user1 First Sexually Transmitted Case Of Zika Virus In U.S. Confirmed
	(#3) Infants	CDC^b^ reports 157 cases of U.S. pregnant women infected with Zika virus.
	(#4) Spread	Zika strain from Americas outbreak spreads in Africa for first time: WHO^c^ (Update)
	(#5) Sports	MLB^d^ moves games from Puerto Rico due to Zika concerns....uh..what about the Olympics?? Can’t be good.
Treatment	(#1) Lack of treatment	RT @DrFriedenCDC: Much is still unknown about #Zika and there is no current medicine for treatment or vaccine to prevent the virus.
	(#2) Zika test	Rapid Zika Test Is Introduced by Researchers The test, done with a piece of paper that changes color if the virus...
	(#3) Vaccine development	Researchers discover structure of Zika virus, a key discovery in development of antiviral treatments and vaccines
	(#4) Blood test	Experimental blood test for Zika screening approved
	(#5) Test development	New mouse model leads way for #Zika drug, vaccine tests

^a^RT: ReTweet.

^b^CDC: Center for Disease Control.

^c^WHO: World Health Organization.

^d^MLB: Major League Baseball.

**Table 5:** Symptoms topic modeling results.

Topic	Words	Tweets
(#1) Zika effects	infect, babies, mosquito, cause, microcephaly, symptom, pregnancy	RT^a^ @USATODAYhealth: Zika affects babies even in later stages of pregnancy. Microcephaly seen in babies from moms infected in 6th month
(#2) Brain defects	brain, link, studies, microcephaly, baby, disorder, cause, damage, infect, fetal	Zika Virus May Cause Microcephaly by Hijacking Human Immune Molecule: Fetal brain model provides first clues on how Z...
(#3) Confirmed defects	defect, cause, birth, confirm, health, severe, link, official	Enough conspiracy theories; nature is nasty enough: U.S. health officials confirm Zika cause of severe birth defects
(#4) Scarier than thought	scarier, than, thought, us, official, health, CDC^b^, warn, learn, first	#breakingnews Zika Virus “Scarier Than We First Thought,” Warn US Health Officials
(#5) Initial reports	first, report, death, case, puerto, confirm, rico, cause, colombia, defect	Colombia Reports First Cases of Microcephaly Linked to Zika Virus—Sun Jan 09 15:13:20 EST

^a^RT: ReTweet.

^b^CDC: Center for Disease Control.

## Discussion

The number of tweets and polarity of tweets were similar between male and female Twitter users with the majority of tweets being negative. There was a similar class imbalance in the random sample of labeled tweets and total corpus showing that the gold standard labeled by experts was an accurate representation of the total corpus. The 2-stage classifier performed well for both levels (relevancy and disease characteristics). Finally, the most persistent topics concerning the disease characteristics were uncovered using topic models.

### Sentiment Analysis and Word Polarity

Although a majority of the tweets were categorized as having negative polarity, the percentage of positive tweets was higher than expected. Some examples of tweets that were classified as positive are as follows: “Case report: assoc btw #Zika/teratogenicity strengthened & evidence shows impact on fetus may take time to manifest,” “RT : At recent int’l meeting about , experts exchanged insights, identified knowledge gaps, and agreed on a plan,” and “91,387 Cases of Zika Confirmed in Brazil This Year: Brazil has confirmed 91,387 cases of...” Words such as “strengthened,” “agreed,” and “confirmed” may be why some tweets were being classified as positive. Sentiment analysis is complex as most sentiment analysis tools just use the word “polarities.” However, contextual information needs to be incorporated for topic-specific sentiment analysis in this domain. We are currently looking into this issue but leave detailed analysis and discussion to a future study.

### Classification Analysis

One of the interesting findings of our analysis was the fact that the MNB classifier outperformed the other more popular classifiers in text analytics: random forest (J48) and SVM. According to 1 study [28], this has to do with the class imbalance issue in our dataset, for both the first (relevancy) and second (disease category) stage of our classifier. This also highlights the possible orthogonality of the features used in our study: the unigrams. Specifically, in this dataset, measuring the likelihood of the features in a given class independently outperforms other complex models such as J48 and SVM. This possibly also relates to the fact that the data are less noisy as they have been evaluated by expert annotators. Naive Bayes is one of the simplest classification models available to us, but it is nonetheless among the most effective for this dataset. This result is non intuitive but not surprising when we consider that using text for classification is relatively imprecise compared with other types of data. In datasets with large amounts of error, simpler models are less likely to overfit the data. Hence we recommend that future research on text analytics begin with Naive Bayes and then proceed to using more complex models to see if these actually improve classification accuracy.

### Annotation Observations

One major issue when annotating tweets was what to do about news tweets like this one: “Your Wednesday Briefing: Bernie Sanders, Hillary Clinton, Zika Virus: Here’s what you need to know to start...” The issue was that this does give relevant information about Zika in that it tells what news stations were discussing and what else was going on at the same time as the Zika outbreak. However, the tweet itself does not give any information about Zika symptoms, treatment, transmission, and prevention. The expert annotators indicated that they decided to code these tweets as relevant because they were about Zika, but we did not include them in the disease characteristics annotations as they do not have any information about the disease characteristics that we outlined *a priori* as our domain of inquiry. This said, they did decide to include information about Zika and sporting events because these could be sources of transmission from athletes and fans not taking proper precautions. We recognize, however, that the sporting context may not have been viewed as important had the Olympics not occurred during the same time as our data collection. Any qualitative deductive coding scheme is underlain by specific assumptions and theoretical constraints that can be highly context-specific, and we feel that it is important for research using citizen sensing to incorporate experts who are able to delineate scientifically accepted contextual boundaries for inquiry.

### Topic Modeling

The perplexity plot (Figure 5) indicates that while we could use a larger number of topics for very small improvements, using a number of topics greater than 5 quickly becomes a case of diminishing returns, especially if we choose to use a parsimonious model to represent our data. Moreover, as we wanted to conduct an exploratory analysis of the topics for this study, the results rationalize our choice of 5 topics.

The emergent topics in prevention (need for control and prevent spread, need for money, ways to prevent spread, bill to get funds, and research) were not surprising considering that there has been much discussion about how to prevent Zika, the need for funding to prevent Zika, and the research required to find a cure for Zika as it is an emerging disease. There is also a need to better understand Zika virus, the disease it causes, and ways to combat it [29]. Looking at the tweets for topics #1 and #4 in transmission, both highlight the concerns and risks associated with Zika spread, which is most likely why they both overlap. Sports was most likely a topic because the tweets were collected during baseball season and just before the Olympics, and many athletes were concerned about getting infected with Zika while competing in the 2016 Olympics in Rio de Janeiro.

In symptoms, topics #1, #3, and #5 were closely related in that they addressed the defects caused by Zika, but nonetheless point to slightly different concepts (Table 5). For example, microcephaly is not the only defect; there is also Guillain-Barré, which would be topic #1. Topic #2 focuses on microcephaly because that is perhaps the most persistent concern related to Zika. Such discussion is seen in these tweets: “RT @USATODAYhealth: Zika affects babies even in later stages of pregnancy. Microcephaly seen in babies from moms infected in 6th month” and “Zika Virus May Cause Microcephaly by Hijacking Human Immune Molecule: Fetal brain model provides first clues on how Z...” Topic #3 contains tweets that occurred when the defects were confirmed to be caused by Zika and not something else: “Zika linked to fetal brain damage: Finnish study: infectious Zika virus from fetal tissue in cell culture. The virus,” and “Enough conspiracy theories; nature is nasty enough: U.S. health officials confirm Zika cause of severe birth defects.”

Topic #4 for symptoms was primarily generated through discussion of a British Broadcasting Corporation article [30] on how more birth defects have been linked to Zika and that the virus was expected to travel further than initially thought, leading to experts admitting that Zika is scarier than was first thought. The statement of Zika being “scarier than we first thought” by the CDC was a big topic on Twitter: “CDC says zika virus scarier than thought as US prepares for outbreak: On Monday, the U.S. Centers for Disease....” This also affected the US political environment: “#2016elections U.S. Officials Warn Zika Scarier Than Initially Thought: By Timothy Gardner and Jeff Mason WA...” This discussion led to additional tweets about the danger of Zika virus: “The Edge: Zika Is Now Even More Terrifying,” “Zika virus ‘shrinks brains’ in tests,” and “#Zika Survivor Says ‘I Could Feel My Skin Shrinking’ CBS Boston.” These tweets demonstrate how a statement by the CDC can be spread and how users can tweak the wording of these CDC statements to generate more concern than is warranted by the actual impact of the disease. Finally, topic #5 includes tweets about initial reports of Zika outbreaks and deaths.

Within symptoms, several tweets in topic #1 were calling Zika a hoax, “Zika HOAX exposed by South American doctors: Brain deformations caused by larvicide chemical,” “The Zika Virus is a hoax! It is like calling the common cold an epidemic. It’s what they put in the drinking water,” and “CDC likely fabricating link between Zika virus and microcephaly cases.” However, the CDC has stated multiple times that Zika and microcephaly are definitely linked: “CDC: Zika definitely causes severe birth defects” and “Here’s a #Zika basic: Zika infection during pregnancy can cause some severe birth defects.” Some of the people saying Zika is a hoax are misunderstanding this quote from the CDC: “People usually don’t get sick enough to go to the hospital, and they very rarely die of Zika. For this reason, many people might not realize they have been infected. Once a person has been infected, he or she is likely protected from future infections.” This statement is true for the majority of healthy adults. However, for infants it can cause microcephaly and in some cases Guillain-Barré syndrome in healthy adults: “Symptoms of Guillain-Barré syndrome include weaknesses in arms & legs. GBS is linked w/ #Zika.” There also have already been multiple deaths due to Zika as was detailed in topic #5. The CDC has also been directly answering questions about Zika on Twitter. One user tweeted at the CDC, “Why is of particular concern to women who are pregnant or considering becoming pregnant? ,” to which the CDC responded, “Zika infection in pregnancy can cause microcephaly and other severe brain defects. .” This shows that while some misinformation is still getting tweeted, the CDC is working to get the correct information out there. This is useful because it shows that the CDC could potentially target specific user groups directly through our classifier and topic modeling approach, and respond to users within a topic group with a similar response that can allow correct information to get transmitted to a larger population with less effort.

All of the topics under the different disease characteristics fit the characteristic. For example, control, money need, prevention, bill, and research were all major topics of prevention discussions. This indicates that the classification model accurately labeled tweets. It also indicates that tweets about major topics were collected and accurately reflected in our topic model. Also, whereas all 4 disease characteristics are important, symptoms was discussed in detail because the researchers felt it included the most important information for public health officials to know especially once the misconceptions and misinformation, such as Zika being a hoax, were found. Categorizing the symptoms into the different topics using topic modeling also allowed us to get deeper into the themes within the symptoms category. This can allow a more targeted interaction with agencies like CDC and specific users to provide interventions against the spread of misinformation. If we are able to make the persistent misconceptions that people have about Zika clear, then public health agencies can inform accordingly.

### Limitations

Although we feel that our methods and findings are trustworthy and robust, we would like to point out some limitations we face in our dataset, and the use of social media.

#### Language Constraint

We have restricted our study to English-language tweets, which certainly limits the strength of our study. This is more critical to address given that South American countries were initially affected by Zika. This also restricts our analysis of measuring disease outbreak, which is why we refrained from doing so in our study. Future studies could address this limitation through analysis of tweets written in Spanish or Portuguese.

#### Keyword Constraint

As described in the Data Collection section, we used the keywords *Zika*, *Zika virus*, *Zika treatment*, and *Zika virus treatment* in our study. Hence, we can expect that this search would overlook tweets that referred to the disease in a different name or talked about the disease without using the word Zika. The keywords *Zika treatment* and *Zika virus treatment* were added because there were few tweets about the treatment of Zika. This was not surprising as there is currently no treatment for Zika. By including those 2 keywords, researchers could download relevant tweets containing those keywords. This was done because we still felt that treatment needed to be included because there was ongoing drug and vaccine research being implemented during the time of tweet collection. From a preliminary manual data analysis, we observed that the other category titles did not need to be included as keywords because more than enough tweets were being collected for those categories. One interesting observation here is that although the keyword “treatment” was part of the crawling process, the treatment subcategory was still the smallest class in the distribution of the dataset (see Figures 3 and 4).

#### Gender and Polarity Constraint

Only 51.49% (635,660/1,234,605) of the tweets were labeled by the gender API using the profile name (Figure 2). Similarly, 11.14% (137,536/1,234,605) of the tweets were not labeled on their polarity. Given the concerns about infant microcephaly and sexual transmission, gender is an important factor to consider when contextualizing discussions around Zika. Gender needs to be addressed moving forward with this study by creating a customized gender recognition tool using machine learning specifically for Twitter data.

### Conclusion

The proportion of tweets between male and female Twitter users was similar by number of tweets in general and by polarity. The majority of tweets were negative but there were more positive tweets than expected, which may be due to the use of positive words such as “strengthened,” “agreed,” and “confirmed.” There was a class imbalance in the ground truth and overall tweets; however, the imbalance was similar between the two, showing that the tweets used in the ground truth were a good representation of the tweets overall. There were hardly any tweets about treatment, which was not surprising because there is no treatment for Zika. The classification performance was very high for relevancy (*F*=0.86) and disease characteristics (*F*=0.94) for the ground truth (*F*=0.99) and for the overall tweets (*F*=0.90). The 5 topics for prevention were control, money need, prevention, bill, and research. The 5 topics for transmission were vectors (mosquitoes), sexual transmission, infants, spread, and sports. The 5 topics for treatment were lack of treatment, Zika test, vaccine development, blood test, and test development. Finally, the 5 topics for symptoms were Zika effects, brain defects, confirmed defects, scarier than thought, and initial reports.

This is one of the first studies to report successful creation of an automated content classification tool to analyze Zika-related tweets, specifically in the area of epidemiology. Through citizen sensing, such a system will help advance the field’s technological and methodological capabilities to harness social media sources for disease surveillance research.

### Future Work

Future studies should include creation and evaluation of an automated technique to detect misinformation using tweets to allow for well-targeted, timely interventions. Such a platform will generate data on emerging temporal trends for more timely interventions and policy responses to misinformation on Zika. We would encourage such studies to leverage multiple information sources including blogs, news articles, as well as social media.

## Abbreviations

API: application program interface
AUC: area under the curve
CDC: Center for Disease Control
DC: disease classifier
GBS: Guillain-Barré syndrome
J48: decision tree
LDA: latent Dirichlet allocation
MNB: Multinomial Naive Bayes
RC: relevancy classifier
SMO: sequential minimal optimization
SVM: support vector machine
WHO: World Health Organization
